# Design, Synthesis, Characterization, and Computational Studies on Benzamide Substituted Mannich Bases as Novel, Potential Antibacterial Agents

**DOI:** 10.1155/2014/732141

**Published:** 2014-01-19

**Authors:** Suman Bala, Neha Sharma, Anu Kajal, Sunil Kamboj

**Affiliations:** M. M. College of Pharmacy, Maharishi Markandeshwar University, Mullana, Ambala, Haryana 133207, India

## Abstract

A series of benzamide substituted Mannich bases **(1–7)** were synthesized. The synthesized derivatives were authenticated by TLC, UV-Visible, FTIR, NMR, and mass spectroscopic techniques and further screened for *in vitro* antibacterial activity by test tube dilution method using amoxicillin and cefixime as standard drugs. The compounds **5**, **6**, and **7** were found to be the most active antibacterial agents among all the synthesized compounds. The physicochemical similarity of the compounds with standard drugs was assessed by calculating various physicochemical properties using software programs. The percent similarity of synthesized compounds was found to be good and compound **1** was found to have higher percentage of similarity. The compounds were subjected to QSAR by multilinear regression using Analyze it version 3.0 software, and four statistically sound models were developed with *R* ^2^ (0.963–0.997), *R*_adj_^2^ (0.529–0.982), and *Q* ^2^ (0.998–0.999) with good *F * (2.35–65.56) values.

## 1. Introduction

The infectious diseases are widely managed by the antimicrobial agents but increase in resistance of microorganisms towards antimicrobial agents in the past few years has become a serious health care problem, and this has led to the necessity of designing of some novel, potent, and safe antimicrobial agents against resistant microbial strains. The compounds having Mannich bases are found to have broad spectrum activity against all strains resistant to other drugs. Mannich bases have gained importance due to their application in antibacterial activity [1–3].

Mannich bases are the end products of Mannich reaction with basic moiety of beta amino ketone [4, 5]. Mannich bases are formed by the condensation of a compound with active hydrogen(s) with an amine (primary or secondary) and formaldehyde (any aldehyde) [6].

Computational studies are the starting point which can be used to predict the experimental data of entirely known molecule or to explore reaction mechanisms and so forth. There are several areas of computational studies and one of them is identification of correlations between chemical structures and properties and is known as QSAR. Quantitative structural activity relationship uses molecular parameters to quantify a pharmacological or chemical property for a set of molecules [7, 8].

In the present work, novel series of seven benzamide substituted Mannich bases are prepared and further screened for the antibacterial activity against Gram negative bacteria (*Escherichia coli, Pseudomonas aeruginosa*) and Gram positive bacteria (*Enterococcus faecalis, Staphylococcus aureus*). Computational studies including QSAR and molecular structural similarity studies were also performed to establish a relationship between biological activities in terms of minimum inhibitory concentration with various physicochemical parameters using multilinear regression.

## 2. Material and Method

### 2.1. Chemistry

TLC plates of 3 × 15 cm coated with silica gel *G* were used for reaction monitoring and for determination of retardation factor. Spots of TLC were located by iodine chamber. Melting points of newly synthesized benzamide substituted Mannich bases were determined on digital melting point apparatus (Flora; Perfit India) and were found uncorrected (Table 1). The structures of the synthesized derivatives were confirmed by spectral data. The *λ*_max_ was calculated by using double beam UV-Visible 1800 Shimadzu spectrophotometer. The IR spectra were recorded on FTIR-Shimadzu spectrometer using Nujol method. ^1^H NMR and ^13^C NMR spectra were recorded on BRUKER AVANCE II NMR spectrometer using DMSO as solvent and TMS as internal standard; chemical shift values were expressed in *δ* ppm. For mass spectra, solutions were made in HPLC grade methanol and spectra were obtained with Vg-11-250 J70S spectrophotometer at 70 eV using electron ionization (EI source). Chem3D Ultra (version 10) was employed for structural similarity studies [9]. QSAR studies were performed by multilinear regression using Analyze it version 3.0 software.

#### 2.1.1. Synthesis

**Figure 1:**
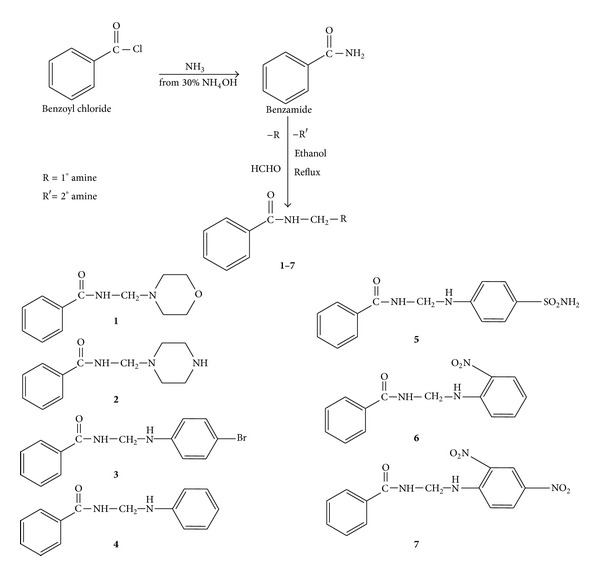
Synthesis of benzamide substituted Mannich bases (**1**–**7**).

A series of benzamide substituted Mannich bases were synthesized as per Figure 1.

*General Procedure for the Synthesis of Benzamide*. To 1 mL of benzoyl chloride, 2 mL of ammonium hydroxide (30%) was added. This mixture was allowed to stand for 2 minutes and then 4 mL water was added to it. The crude product was recrystallized with ethanol [10].

*N-(Morpholin-4-ylmethyl)benzamide *(**1**)* [11, 12]*. Benzamide (0.001 M) was dissolved in ethanol. To this morpholine (0.001 M) and (0.015 M) formaldehyde were added. The reaction mixture was refluxed for 1 hour and the reaction was monitored by TLC. Light brown colored crystals were obtained after evaporating the solvent. Mp 65–67°C; IR (Nujol): 3428.13, 3050.47, 2828.85, 1655.23, 1543.20, 1539.42, 1432.65, 1309.87, 1277.90, 1058.66, 1012.79, 817.81 cm^−1^; ^1^H NMR (DMSO-d_6_ 400 MHz): 8.0 (br, s, 1H, –NH), 7.95 (m, 2H, Ar–H), 7.51 (m, 3H, Ar–H), 4.27 (s, 2H, –CH_2_), 3.67 (s, 4H, –H of morpholine), 2.37 (s, 4H, –H of morpholine); ^13^C NMR: 71.5 (C-2,6), 53.9 (C-3,5), 64 (C-7), 167.4 (C-9), 132.9 (C-11), 127.6 (C-12,16), 131.9 (C-13,14,15); MS: *m/z* 220.12, 221.10 (M+1), 222.13 (M+2); Anal. Cal. for C_12_H_16_N_2_O_2_: C: 65.43, H: 7.32, N: 12.72, O: 14.53. Found: C: 65.23, H: 7.39, N: 12.12, O: 14.33. 

*N-(Piperazine-1-ylmethyl)benzamide* (**2**)  * [13]*. Benzamide (0.001 M) was dissolved in ethanol. To this piperazine (0.001 M) and (0.015 M) formaldehyde were added. The reaction mixture was refluxed for 7-8 hours and the reaction was monitored by TLC. Dark brown colored crystals were obtained after evaporating the solvent. Mp 68–70°C; IR (Nujol): 3414.15, 3053.45, 2839.88, 1648.24, 1567.23, 1534.44, 1433.66, 1306.83, 1012.73, 818.82 cm^−1^; ^1^H NMR (DMSO-d_6_ 400 MHz): 8.4 (br, s, 1H, –NH), 7.98 (m, 2H, Ar–H), 7.52 (m, 3H, Ar–H), 4.26 (s, 2H, –CH_2_), 2.65 (s, 4H, –H of piperazine), 2.48 (s, 4H, –H of piperazine), 2.0 (br, s, 1H, –NH); ^13^C NMR: 50.8 (C-2,6), 55.1 (C-3,5), 64.1 (C-7), 168.2 (C-9), 133.2 (C-11), 127.9 (C-12,16), 130.9 (C-13,14,15); MS: *m/z* 219.14, 220.14 (M+1), 221.17 (M+2); Anal. Cal. for C_12_H_17_N_3_O: C: 65.73, H: 7.81, N: 19.16, O: 7.30. Found: C: 65.79, H: 7.61, N: 19.26, O: 7.32. 

*N-[(4-Bromo-phenylamino)-methyl]-benzamide* (**3**). Benzamide (0.001 M) was dissolved in ethanol. To this 4-bromoaniline (0.001 M) and (0.015 M) formaldehyde were added. The reaction mixture was refluxed for 9-10 hours and the reaction was monitored by TLC. Light orange colored crystals were obtained after evaporating the solvent. Mp 66–69°C; IR (Nujol): 3439.23, 3052.48, 2834.52, 1645.25, 1538.30, 1502.61, 1460.15, 1094.64, 1015.57, 852.57 cm^−1^; ^1^H NMR (DMSO-d_6_ 400 MHz): 8.2 (br, s, 1H, –NH), 7.95 (m, 2H, Ar–H), 7.56 (m, 3H, Ar–H), 7.21 (d, 2H, Ar–H), 6.32 (d, 2H, Ar–H), 4.97 (t, 2H, –CH_2_), 4.0 (br, s, 1H, –NH); ^13^C NMR: 38.6 (C-1), 30.8 (C-2,6), 29.6 (C-3,5), 47.9 (C-4), 57.8 (C-9), 168.2 (C-11), 133.5 (C-13), 127.3 (C-14,18), 129.7 (C-15,16,17); MS: *m/z* 304.02, 306.02 (M+1), 305.01 (M+2); Anal. Cal. for C_14_H_13_BrN_2_O: C: 55.10, H: 4.29, Br: 26.18, N: 9.18, O: 5.24. Found: C: 55.18, H: 4.19, Br: 26.28, N: 9.08, O: 5.44. 

*N-Phenyl-aminomethyl-benzamide* (**4**). Benzamide (0.001 M) was dissolved in ethanol. To this aniline (0.001 M) and (0.015 M) formaldehyde were added. The reaction mixture was refluxed for 9-10 hours and the reaction was monitored by TLC. Light brown colored crystals were obtained after evaporating the solvent. Mp 98–101°C; IR (Nujol): 3442.12, 3054.41, 2836.59, 1652.10, 1539.26, 1504.54, 1440.28, 1013.64, 822.68 cm^−1^; ^1^H NMR (DMSO-d_6_ 400 MHz): 8.1 (br, s, 1H, –NH), 7.98 (m, 2H, Ar–H), 7.52 (m, 3H, Ar–H), 6.58 (m, 2H, Ar–H), 7.04 (m, 3H, Ar–H), 4.96 (s, 2H, –CH_2_), 4.2 (br, s, 1H, –NH); ^13^C NMR: 48.6 (C-1), 33.4 (C-2,6), 24.3 (C-3,4,5), 57.8 (C-8), 168.4 (C-10), 133.0 (C-12), 127.6 (C-13,17), 131.2 (C-14,15,16); MS: *m/z* 226.11, 227.13 (M+1), 228.12 (M+2); Anal. Cal. for C_14_H_14_N_2_O: C: 74.31, H: 6.24, N: 12.38, O: 7.07. Found: C: 74.38, H: 6.04, N: 12.39, O: 7.13. 

*N-[(4-Sulfamoyl-phenylamino)-methyl]-benzamide* (**5**). Benzamide (0.001 M) was dissolved in ethanol. To this sulfanilamide (0.001 M) and (0.015 M) formaldehyde were added. The reaction mixture was refluxed for 10–12 hours and the reaction was monitored by TLC. Colorless crystals were obtained after evaporating the solvent. Mp 69-70°C; IR (Nujol): 3310.16, 3054.41, 2854.69, 1636.52, 1541.19, 1534.44, 1440.82, 1320.22, 1135.55, 1080.18, 863.18 cm^−1^; 8.2 (br, s, 1H, –NH), 7.96 (m, 2H, Ar–H), 7.71 (d, 2H, Ar–H), 7.51 (m, 3H, Ar–H), 6.71 (d, 2H, Ar–H), 4.94 (t, 2H, –CH_2_), 4.0 (br, s,1H, –NH), 2.0 (br, s, 2H, –NH_2_); ^1^H NMR (DMSO-d_6_ 400 MHz): 8.1 (br, s, 1H, –NH), 7.98 (m, 2H, Ar–H), 7.52 (m, 3H, Ar–H), 6.58 (m, 2H, Ar–H), 7.04 (m, 3H, Ar–H), 4.96 (s, 2H, –CH_2_), 4.2 (br, s,  1H, –NH); ^13^C NMR: 58.4 (C-1), 15.7 (C-2,6), 29.5 (C-3,5), 48.6 (C-4), 57.4 (C-10), 167.9 (C-12), 132.9 (C-16), 127.6 (17,21), 130.4 (C-18,19,20); MS: *m/z* 305.09, 306.10 (M+1), 307.08 (M+2); Anal. Cal. for C_14_H_15_N_3_O_3_S: C: 55.07, H: 4.95, N: 13.76, O: 15.72, S: 10.50. Found: C: 55.13, H: 4.75, N: 13.26, O: 15.79, S: 10.42. 

*N-[(2-Nitro-phenylamino)-methyl]-benzamide*  (**6**). Benzamide (0.001 M) was dissolved in ethanol. To this 2-nitroaniline (0.001 M) and (0.015 M) formaldehyde were added. The reaction mixture was refluxed for 10–12 hours and the reaction was monitored by TLC. Yellow colored crystals were obtained after evaporating the solvent. Mp 68–70°C; IR (Nujol): 3423.80, 3050.21, 2831.05, 1646.32, 1540.19, 1524.36, 1509.36, 1462.21, 1098.51, 1306.55, 859.32 cm^−1^; ^1^H NMR (DMSO-d_6_ 400 MHz): 8.4 (br, s, 1H, –NH), 7.97 (m, 1H, Ar–H), 7.95 (m, 2H, Ar–H), 7.54 (m, 3H, Ar–H), 7.43 (m, 1H, Ar–H), 6.84 (m, 1H, Ar–H), 6.69 (m, 1H, Ar–H), 4.99 (s, 2H, –CH_2_), 4.21 (br, s, 1H, –NH); ^13^CNMR: 46.9 (C-1), 28.8 (C-2), 21.3 (21.3), 22.5 (C-4), 22.9 (C-5), 85.3 (C-6), 129.4 (C-15,16,17); MS: *m/z* 271.10, 272.10 (M+1), 273.15 (M+2); Anal. Cal. for C_14_H_13_N_3_O_5_: C: 61.99, H: 4.83, N: 15.49, O: 17.69. Found: C: 61.91, H: 4.80, N: 15.55, O: 17.42. 

*N-[(2,4-Dinitro-phenylamino)-methyl]-benzamide* (**7**). Benzamide (0.001 M) was dissolved in ethanol. To this 2,4-dinitroaniline (0.001 M) and (0.015 M) formaldehyde were added. The reaction mixture was refluxed for 10–12 hours and the reaction was monitored by TLC. Yellow colored crystals were obtained after evaporating the solvent. Mp 69–72°C; IR (Nujol): 3426.80, 3052.21, 2835.05, 1641.32, 1545.19, 1530.36, 1502.36, 1466.21, 1099.51, 1304.55, 862.32 cm^−1^; ^1^H NMR (DMSO-d_6_ 400 MHz): 8.90 (m, 1H, Ar–H), 8.36 (m, 1H, Ar–H), 8.2 (br, s, 1H, –NH), 7.95 (m, 2H, Ar–H), 7.5 (m, 3H, Ar–H), 6.95 (m, 1H, Ar–H), 4.94 (s, 2H, –CH_2_), 4.1 (br, s, 1H, –NH); ^13^C NMR; 45.9 (C-1), 24.2 (C-2), 21.9 (C-3), 76.7 (C-4), 23.5 (C-5), 80.7 (C-6), 56.7 (C-8), 167.9 (C-10), 133.2 (C-15), 127.9 (C-16,20), 130.2 (C-17,18,19); MS: *m/z* 316.09, 317.10 (M+1), 318.10 (M+2); Anal. Cal. for C_14_H_12_N_4_O_5_: C: 53.17, H: 3.82, N: 17.71, O: 25.29. Found: C: 53.23, H: 3.88, N: 17.72, O: 25.18. 

## 3. Biological Evaluation

### 3.1. Antibacterial Activity

The inhibition of the microbial growth may be utilized for demonstrating the therapeutic efficacy of the synthesized compounds. The Gram negative bacteria (*Escherichia coli, Pseudomonas aeruginosa*) and Gram positive bacteria  (*Enterococcus faecalis, Staphylococcus aureus*) were used for the activity.

The antibacterial activity was evaluated by tube dilution method which depends upon the inhibition of growth of a microbial culture in a uniform solution of antibiotic in a fluid medium that is favorable to its rapid growth in the absence of the antibiotic [14]. In this method minimum inhibitory concentration (MIC) of the test compounds was determined. The MIC is the lowest concentration of an antimicrobial agent that inhibits the growth of the test organism [15]. Test compounds and standard compounds (amoxicillin and cefixime) were dissolved in dimethyl sulfoxide to give a concentration of 100 *μ*g/mL. Double strength nutrient broth I.P. was used. Suspension of each microorganism was made by transferring the organism from culture to 10 mL of sterile normal saline solution. 

**Figure 2:**
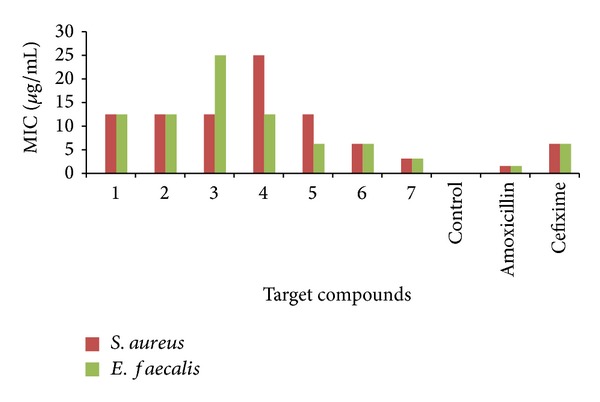
Graphical representation of minimum inhibitory concentration (MIC) of benzamide substituted Mannich bases against Gram positive bacterial strains.

**Figure 3:**
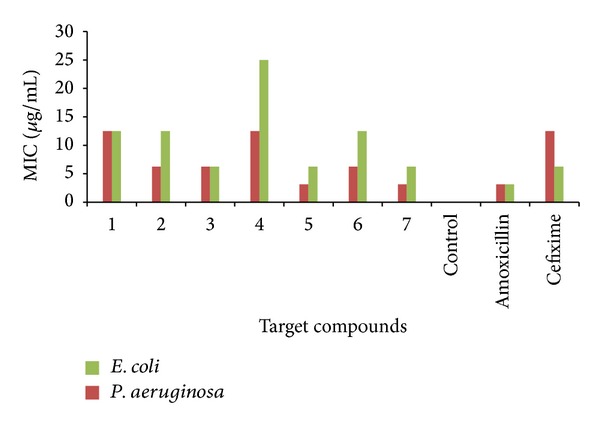
Graphical representation of minimum inhibitory concentration (MIC) of benzamide substituted Mannich bases against Gram negative bacterial strains.

*Determination of Minimum Inhibitory Concentration (MIC)*. One mL of sterilized media was poured into sterile test tubes. One mL of 100 *μ*g/mL test solution was transferred in one tube and serially diluted to give concentrations of 50, 25, 12.5, 6.25, 3.12, 1.56, and 0.78 *μ*g/mL. To all the tubes 0.1 mL of suspension of bacteria in saline was added and the tubes were incubated at 37°C for 24 h. The growth in the tube was observed visually for turbidity and inhibition was determined by the absence of growth. MIC was determined by the lowest concentration of sample that retarded the development of turbidity. The activity of the compounds compared with the standard drugs (amoxicillin and cefixime) is given in Table 2. Graphical representation of minimum inhibitory concentration (MIC) of benzamide substituted Mannich bases against Gram positive and negative bacterial strains is given in Figures 2 and 3, respectively. 

## 4. Molecular Structural Similarity Studies

Assessments of molecular structural similarity of synthesized benzamide substituted Mannich bases were compared to those of standard compounds by means of physicochemical similarity between the standard drugs and new analogues designed. The information was used for prediction of biological activity of important target compounds for novel drug discovery. The physicochemical parameters were calculated for the synthesized compounds using Chem3D Ultra (version 10) and compared with the values obtained for standard compounds (cefixime and tosufloxacin tosylate). Various set of parameters were used for calculations as given in Table 3.

The distance *d*_*i*_ of a particular target compound *i* can be presented as

(1) di2=∑j=1n(1−Xi,j/Xi,standard)2n,

where *X*_*i*,*j*_ is the value of molecular parameters *i* for compound *j*.

*X*_*i*,standard_ is the value of the same molecular parameter *i* for standard drug.

*n* is the total number of the considered molecular parameters.

The similarity of the compounds can be calculated as

(2) $$\begin{array}{c} \%\;\text{age}\;\text{similarity}=\left(1-R\right)\times 100, \end{array}$$

where *R* is quadratic mean also known as the root mean square and *R* can be calculated as

(3) $$\begin{array}{c} R=\sqrt{d_{i}^{2}}. \end{array}$$

Assessment of structural similarities of synthesized compounds with standard drugs showed that all the derivatives have good % age similarity (Table 4).

## 5. Quantitative Structure Activity Relationship (QSAR)

The synthesized substituted Mannich bases were subjected to QSAR analysis by using multilinear regression. For this analysis, various physicochemical parameters were calculated and correlated with biological activity, that is, antibacterial activity to obtain QSAR models. The physicochemical parameters were computed using Chem3D (Version 10) Ultra after energy minimization to minimum root mean square (RMS) gradient of 0.100 kcal/mole Å by MOPAC software package. Out of all the physicochemical parameters (Table 4), the following five parameters were selected for QSAR studies: log *P*, Connolly solvent accessible surface area (SAS), molar refractivity (MR), ovality, and molecular weight (MW). Biological activity data was converted to the logarithmic values. For antibacterial activity, biological activity was taken as −log(MIC/molecular weight of compound).

### 5.1. Statistical Analysis

The statistical significance of the models was assessed on the basis of various parameters such as *R* ^2^ (coefficient of correlation), *R*_adj_^2^ (coefficient of determination), *Q* ^2^ (cross validates *R* ^2^), and *F* (Fischer statistics), considering all the parameters in the model significant only at 95% confidence level (*P* < 0.05).

**Scheme 1:**
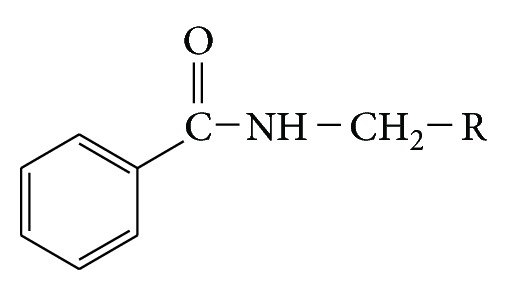


Basic structure of synthesized benzamide substituted Mannich bases is shown in Scheme 1.

*(I) QSAR Model for Antibacterial Activity against Escherichia coli:*

(4) pMIC=0.06413(logP)−0.009411(SAS)−0.003046(MW)+8.615(Ovality)  −0.02413(MR)−4.57,N=7,  R2=0.921,    Radj2=0.529,Press=0.00000025,  Q2=0.999,  F=2.35,  S=0.188.

Here and hereafter,  *R* ^2^: is coefficient of correlation, *R*_adj_^2^: is coefficient of determination, *F*: is Fischer statistics, *N*: is number of test compounds, Press: is predictive error sum of squares, *Q* ^2^: is cross validated *R* ^2^, BA: is biological activity, and *S*: is standard error of estimation.

**Figure 4:**
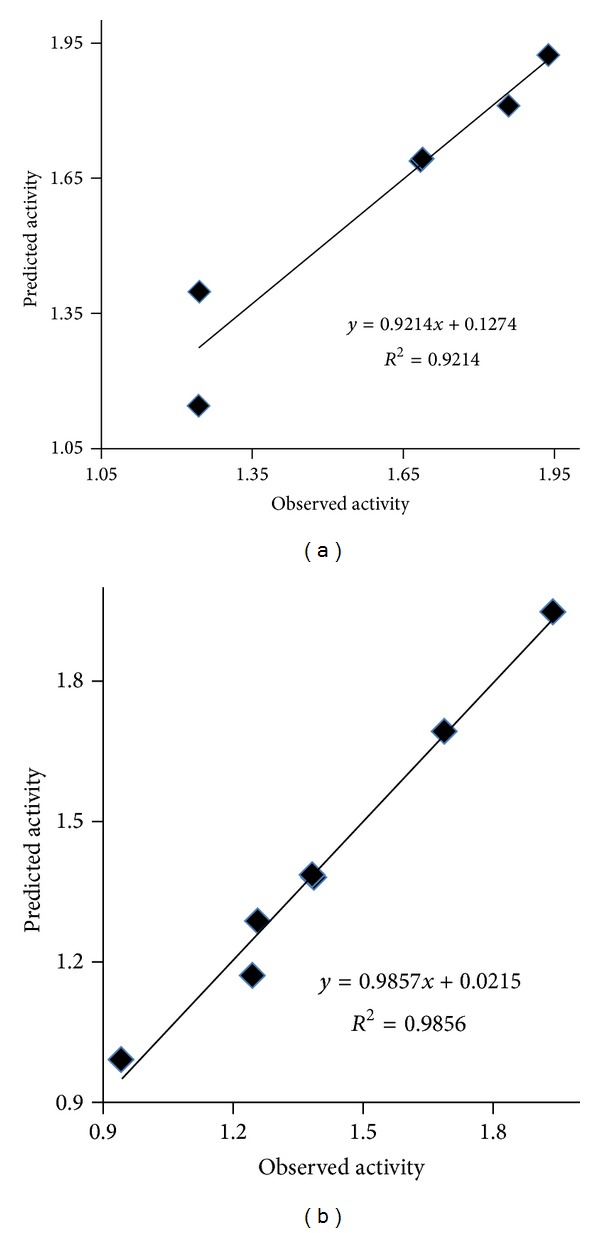
Plot of Predicted pMIC values against observed pMIC for QSAR model for (a) *Escherichia coli* and (b) *Staphylococcus aureus.*

The observed and predicted antibacterial activity of synthesized benzamide substituted Mannich bases are summarized in Table 5 and plot of calculated and observed antibacterial activity is given in Figure 4. 

*(II) QSAR Model for Antibacterial Activity against Staphylococcus aureus:*

(5) pMIC=0.06698(logP)−0.02004(SAS)+0.003104(MW)+5.643(Ovality)−0.04099(MR)+3.876,N=7,  R2=986,  Radj2=0.913,  Press=0.00094,  Q2  =  0.999,    F  =  13.66,  S  =  0.095.

The observed and predicted antibacterial activity of synthesized benzamide substituted Mannich bases are summarized in Table 6 and plot of calculated and observed antibacterial activity is given in Figure 4. 

*(III) QSAR Model for Antibacterial Activity against Pseudomonas aeruginosa:*

(6) pMIC=0.08457(logP)−0.008497(SAS)−0.006487(MW)+7.007(Ovality)−0.03631(MR)−1.385,N=7,  R2  =  0.997,    Radj2=0.982,Press=0.00009,  Q2=0.998, F=65.56,    S=0.034.

**Figure 5:**
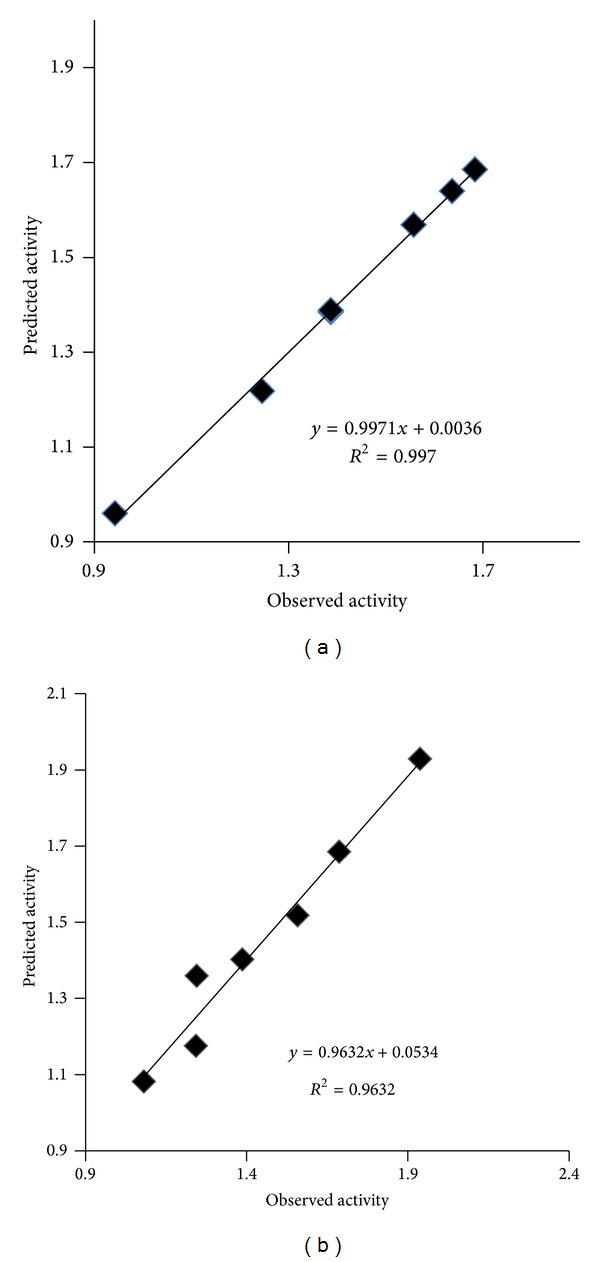
Plot of Predicted pMIC values against observed pMIC for QSAR model for (a) *Pseudomonas aeruginosa* and (b) *Enterococcus faecalis.*

The observed and predicted antibacterial activity of synthesized benzamide substituted Mannich bases are summarized in Table 7 and plot of calculated and observed antibacterial activity is given in Figure 5. 

*(IV) QSAR Model for Antibacterial Activity against Enterococcus faecalis:*

(7) pMIC=0.0383(logP)−0.02271(SAS)+0.002366(M.W)+8.135(Ovality)−0.03458(MR)+1.51,N=7,  R2=0.963,  Radj2=0.779,  Press=0.00000009,  Q2=0.998,  F=5.23,    S=0.139.

The observed and predicted antibacterial activity of synthesized benzamide substituted Mannich bases are summarized in Table 8 and plot of calculated and observed antibacterial activity is given in Figure 5.

## 6. Result and Discussion

### 6.1. Chemistry

The structures of synthesized derivatives were supported by means of chromatographic and spectroscopic methods. Both analytical and spectral data (IR and ^1^H NMR) of all the synthesized derivatives were in full agreement with the proposed structures. The structures of the synthesized derivatives were proven by the spectroscopic method. The structures assigned to (**1**–**7**) were supported by IR spectra showing absorption bands at 3,442–3,310 cm^−1^ due to N–H stretching. Carbonyl stretch was observed at 1,655–1,636 cm^−1^. Stretching vibrations due to C=C (aromatic) were observed at 1,539–1,502 cm^−1^, respectively. Bands at 1,509–1,502 cm^−1^ appeared due to asymmetric N=O stretch. Bands at 863-817 cm^−1^ indicated out-of-plane aromatic stretch. The proton NMR of these compounds revealed the presence of downfield singlet 8.0–8.9 ppm for –CONH. A 2 protons singlet at 4.27–4.99 ppm appeared due to protons of methylene group. All the other aliphatic and aromatic protons were observed within the expected regions. This part concluded the synthesis of benzamide substituted Mannich bases.

### 6.2. Biological Activity

#### 6.2.1. Antibacterial Activity

The minimum inhibitory concentrations of **5 **(12.5, 6.25, 3.125, and 6.25), **6 **(6.25, 6.25, 6.25, and 12.5), and **7 **(3.125, 3.125, 3.125, and 6.25) were found to be good which is comparable with MIC of both standard drugs amoxicillin (1.56, 1.56, 3.125, and 3.125) and cefixime (6.25, 6.25 12.5, and 6.25). No inhibitory effect was observed for DMSO used as control. The MIC of **7** was found to be better than cefixime and MIC of **6** was found to be comparatively similar to cefixime. While studying MIC against bacterial strains **7 **was found to be most active among all the synthesized benzamide substituted Mannich bases. From the above results it was concluded that compounds bearing substitutions such as sulphonamido **5**, *p-*nitro **6**, and dinitro group **7 **have emerged out as potent antibacterial agents.

The compounds bearing nitro groups were found to have significant potential against all bacterial strains. This activity was attributed to lipophilic nature of these substituents which ultimately facilitate the transportation of target compounds across the biological membranes to produce desired effect. Both nitro and sulphonamide groups are an efficient –I, –R group, but the nature of the latter (low log*P* value) is more hydrophilic than the former (high log*P* value). Hence the contribution of sulphonamide group towards antibacterial activity is comparatively less than compounds bearing nitro groups.

#### 6.2.2. Similarity Studies

Assessment of structural similarities of synthesized benzamide substituted Mannich bases with antibacterial drugs (cefixime and tosufloxacin tosylate) indicated that all the derivatives have shown good percentage of similarity ranging from 52.89% to 97.72% and compound **1** was found to have excellent percentage of similarity (>90%) with all standard drugs. The structural similarity of all the compounds with standard drugs varying from one compound to another. This may be due to the large difference between the values of physicochemical parameters calculated for target compounds with values of parameters calculated for standard drugs. However good structural similarity of compounds does not always lead to good therapeutic activity as compound **1 **has shown an excellent percentage of similarity (52.89–97.72%), but it was found to be less active as antibacterial agent.

### 6.3. Quantitative Structure Activity Relationship (QSAR) Analysis

The results suggested that antibacterial activity was highly dependent on log*P*, SAS, MR, ovality, and MW. All the developed models have good coefficient of correlation (0.963–0.997), coefficient of determination (0.529–0.982), and cross validated *R* ^2^ (0.998–0.999) with Fischer statistics (2.35–65.56). The derived models could be used in designing of more potent inhibitors against bacterial infection. The five parameters correlated; statistically sound QSAR models for antibacterial activity against *Staphylococcus aureus*, *Enterococcus faecalis, Escherichia coli,* and *Pseudomonas aeruginosa* could be used for the prediction of biological activities of unknown and unavailable compounds of this class. In the present study, an attempt has been made to design some active antibacterial agents with lesser side effects. The QSAR results have shown the dependence of antibacterial activity of synthesized substituted benzamide Mannich base derivatives on their structural and physicochemical features. The results indicated that the bulkier aromatic substituents like sulphonamido group may increase lipophilicity and give better antibacterial activity. The presence of electron withdrawing group like –NO_2_ in the compound resulted in enhanced biological activity by improving lipophilicity. This lipophilicity could facilitate penetration or passage of these compounds across the biological membrane easily for beneficial therapeutic effect.

## 7. Conclusion

In antibacterial activity, the compounds **5**, **6**, and **7 **were observed as good antibacterial agents and compound **7 **was found to be the most active against all the selected bacterial strains. The compound **1 **was found to have excellent percentage of similarity (>90%) with all standard drugs. The results suggested that antibacterial activity is highly dependent on physicochemical parameters such as log*P*, Connolly solvent accessible surface area (SAS), molar refractivity (MR), ovality, and molecular weight (MW). The derived model could be used in the future for designing of more potent inhibitors of bacterial infection.

## Figures and Tables

**Table 1:** Physical data of synthesized benzamide substituted Mannich bases.

Compound	Color	Solubility	*λ*_max_	*R*_*f*_ value^#^	% age yield	Molecular weight
1	Light brown crystals	DMSO, EtOH, CHCl_3_	226	0.45	84	220.273
2	Dark brown crystals	DMSO, EtOH, CHCl_3_	324	0.61	78.2	219.288
3	Light orange crystals	DMSO, EtOH, CHCl_3_	227	0.78	76	305.176
4	Light brown crystals	DMSO, EtOH, CHCl_3_	275	0.71	70.8	226.279
5	Colorless crystals	DMSO, EtOH, CHCl_3_	253	0.65	91.6	302.378
6	Yellow crystals	DMSO, EtOH, CHCl_3_	371	0.82	75	271.277
7	Yellow crystals	DMSO, EtOH, CHCl_3_	346	0.73	80	316.275

^#^Stationary phase: silica gel. Mobile phase for TLC: petroleum ether : ethyl acetate : methanol (6 : 3 : 1). Iodine vapors as visualizing agent.

**Table 2:** Minimum inhibitory concentration (MIC) of the synthesized benzamide substituted Mannich bases against Gram positive and Gram negative bacteria.

Compounds	Minimum inhibitory concentration (MIC) (*μ*g/mL)			
	Gram positive bacteria		Gram negative bacteria	
	*S. aureus *	*E. faecalis *	*E. coli *	*P. aeruginosa *
1	12.5	12.5	12.5	12.5
2	12.5	12.5	6.25	12.5
3	12.5	25	6.25	6.25
4	25	12.5	12.5	25
5	12.5	6.25	3.125	6.25
6	6.25	6.25	6.25	12.5
7	3.125	3.125	3.125	6.25
Control	—	—	—	—
Amoxicillin	1.56	1.56	3.125	3.125
Cefixime	6.25	6.25	12.5	6.25

**Table 3:** Calculation of various molecular properties of the synthesized benzamide substituted Mannich bases.

Compound	SAS^a^ (Å^2^)	MS^b^ (Å^2^)	CSEV^c^ (Å^3^)	Ovality	MR^d^ (cm^3^/mol)	MTI^e^	WI^f^	BI^g^	MW^h^	log *P*
1	448.081	228.85	191.50	1.4243	61.4384	3711	496	89200	220.273	1.0523
2	512.423	268.228	223.94	1.5040	42.9696	8354	1126	343784	305.36	1.484
3	538.939	291.721	262.45	1.4715	30.8167	9773	1244	354337	302.378	4.943
4	443.839	227.461	194.33	1.4019	63.1193	3795	496	89200	219.288	0.8303
5	464.20	234.624	187.75	1.4796	51.6276	4798	614	124234	226.278	2.7389
6	483.994	250.613	208.85	1.4721	48.2504	5338	727	164356	305.176	3.5678
7	470.56	247.467	212.78	1.4357	35.1809	6884	925	256945	271.277	—
Std.1*	580.224	331.272	320.88	1.4614	10.691	15459	2353	1046550	437.46	—
Std.2*	570.267	318.963	282.94	1.5303	9.792	13519	2039	748946	404.350	2.419

^a^Connolly solvent accessible surface area. ^b^Connolly molecular surface area. ^c^Connolly solvent excluded volume. ^d^Molar refractivity.

^e^Molecular topological index. ^f^Wiener index. ^g^Balaban index. ^h^Molecular weight. Std.1*—cefixime, Std.2*—tosufloxacin tosylate.

**Table 4:** Assessment of structural similarity of synthesized benzamide substituted Mannich bases with standard drugs.

Compound	Cefixime (1 − *R*)100	Tosufloxacin tosylate (1 − *R*)100
1	72.7	97.72
2	84	67.9
3	90.34	52.89
4	68.2	66.3
5	95.2	69.5
6	91	54
7	74	96.7

**Table 5:** Observed and predicted antibacterial activity of synthesized benzamide substituted Mannich bases against *Escherichia coli*.

Compound	Observed activity	Predicted activity	Residual
1	1.246051254	1.398072	−0.15202
2	1.68893213	1.692905	−0.00397
3	1.684670173	1.688051	−0.00338
4	1.244104854	1.145049	0.099056
5	1.85979231	1.810427	0.049366
6	1.688673205	1.692826	−0.00415
7	1.938562952	1.922897	0.015666

**Table 6:** Observed and predicted antibacterial activity of synthesized benzamide substituted Mannich bases against *Staphylococcus aureus*.

Compound	Observed activity	Predicted activity	Residual
1	1.246051254	1.170593	0.07545782
2	1.387902134	1.38014	0.00776234
3	1.38360177	1.386275	−0.0026351
4	0.943074858	0.991585	−0.0485102
5	1.257732318	1.286955	−0.0292232
6	1.688673205	1.692675	−0.0040022
7	1.93925838	1.947837	−0.0085788

**Table 7:** Observed and predicted antibacterial activity of synthesized substituted benzamide Mannich bases against *Pseudomonas aeruginosa*.

Compound	Observed activity	Predicted activity	Residual
1	1.246051	1.217519	0.028532
2	1.387902	1.384015	0.003887
3	1.68467	1.684545	0.000125
4	0.943075	0.959732	−0.01666
5	1.558762	1.568366	−0.0096
6	1.387643	1.388065	−0.00042
7	1.637533	1.639689	−0.00216

**Table 8:** Observed and predicted antibacterial activity of synthesized benzamide substituted Mannich bases against *Enterococcus faecalis*.

Compound	Observed activity	Predicted activity	Residual
1	1.246051	1.358788	−0.11274
2	1.387902	1.401507	−0.0136
3	1.08261	1.0811	0.00151
4	1.244105	1.174548	0.069557
5	1.558762	1.517701	0.041061
6	1.688673	1.684798	0.003875
7	1.939258	1.928613	0.010645
